# Gut–ocular surface axis in dry eye disease: phenotype-specific mechanisms, evidence, and microbiome-targeted interventions

**DOI:** 10.3389/fcimb.2026.1873050

**Published:** 2026-07-06

**Authors:** Zhizi Zhang, Dandan Liao, Lifeng Zhao, Xuejing Lu

**Affiliations:** 1 Eye School of Chengdu University of TCM, Chengdu, China; 2 Sichuan Provincial Key Laboratory of Traditional Chinese Medicine for Ophthalmic Health, Chengdu, China; 3 Retinal Image Technology and Chronic Vascular Disease Prevention & Control and Collaborative Innovation Center, Chengdu, China

**Keywords:** disease heterogeneity, dry eye disease, gut microbiota, gut-eye axis, microbiome intervention, ocular surface homeostasis

## Abstract

Dry eye disease is a multifactorial, heterogeneous ocular surface disorder characterized primarily by an imbalance in tear film homeostasis. The traditional classification into “aqueous deficiency” and “evaporative” types fails to fully account for the differences in its inflammatory biology, clinical manifestations, and treatment responses. In recent years, the gut microbiota has been implicated in influencing ocular surface homeostasis through immune-inflammatory, metabolic-barrier, and neuroimmune pathways; however, the magnitude of its effects and their biological significance may vary depending on the specific phenotype of dry eye disease (DED). This article reviews the current evidence regarding the gut-ocular surface axis in dry eye disease from a phenotype-specific perspective, categorizing it into direct clinical evidence, animal and mechanistic evidence, indirect and inferential evidence, and hypothesis-generating evidence based on the source and directness of the evidence. The existing evidence is primarily focused on Sjögren syndrome-associated and other immune-mediated forms of dry eye disease. Clinical microbiome studies, germ-free animal models, antibiotic-induced dysbiosis models, and patient-derived microbiota transplantation experiments all suggest that gut microbiota dysbiosis may contribute to systemic immune remodeling and lacrimal-ocular surface inflammatory responses. In contrast, for dry eye syndromes dominated by meibomian gland dysfunction or evaporative dry eye, as well as non-Sjögren aqueous-deficient dry eye, current evidence is primarily supported indirectly by studies on metabolic susceptibility, local microbiome, and animal mechanisms; whereas postoperative, environment-related, and symptom-sign incongruence types of dry eye are more often characterized by early clues or research hypotheses related to host inflammatory thresholds, ocular surface repair capacity, and neuroimmune regulation. Although microbiome-targeted interventions (including probiotics, prebiotics, synbiotics, postbiotics, and fecal microbiota transplantation) have demonstrated some translational potential, they remain limited by small sample sizes, high heterogeneity in study designs, short follow-up periods, and a lack of validation through phenotypic stratification. Future research should shift from general descriptions of microbial differences to stratified cohorts, causal validation, functional multi-omics analysis, and mechanism-driven intervention trials to clarify the true role of the gut microbiota in different DED phenotypes and to advance the development of precision adjunctive treatment strategies.

## 1 Introduction

Dry eye disease (DED) is a common chronic ocular surface disorder with a recurrent course and a substantial long-term management burden (Craig et al., 2017). In recent years, the conceptualization of DED has shifted from the traditional view of insufficient tear production or excessive evaporation toward a more comprehensive pathophysiological framework. The TFOS DEWS II report defines DED as a multifactorial ocular surface disease characterized by loss of tear film homeostasis, in which tear film instability, hyperosmolarity, ocular surface inflammation and damage, and neurosensory abnormalities collectively contribute to disease pathogenesis and progression (Craig et al., 2017). Because prevalence estimates vary according to diagnostic criteria, population characteristics, and assessment methods, the reported burden of DED differs substantially across studies; global prevalence has been estimated to reach approximately 34.6%, making DED an important public health concern (Xiao et al., 2025).

However, DED shows substantial interindividual variability in clinical presentation, underlying mechanisms, and treatment response, indicating that traditional classifications do not fully capture its phenotypic complexity. In the Dry Eye Assessment and Management (DREAM) study, latent profile analysis identified five clinical subtypes among patients with moderate-to-severe DED, and these subtypes showed different responses to omega-3 supplementation (Yu et al., 2022). Tear cytokine profiling has also revealed distinct inflammatory subgroups in patients with DED (Chen et al., 2024). These findings suggest that DED heterogeneity extends beyond symptoms and signs to include biologically distinct inflammatory profiles. Therefore, studies of DED-related mechanisms and interventions should account for disease heterogeneity rather than simply pooling patients with different pathological backgrounds.

The gut microbiota has attracted increasing attention for its role in immune regulation, barrier maintenance, and metabolic homeostasis, and research in this field has expanded from gastrointestinal diseases to a broad range of ophthalmic disorders. The gut microbiota may influence distant organs through immune, metabolic, neural, and endocrine pathways, thereby forming the regulatory network commonly referred to as the gut–eye axis (Labetoulle et al., 2024). Among ocular surface diseases, DED has become a major focus of gut–eye axis research. The proposed gut–eye–lacrimal gland–microbiome axis has further expanded current understanding of the relationship between systemic microbial dysbiosis and DED (Trujillo-Vargas et al., 2020). Although microbial dysbiosis may affect ocular surface homeostasis through multiple pathways, its impact is likely to vary across DED phenotypes, providing a systems-level perspective for interpreting differences in inflammatory background, treatment response, and disease progression among patients (Watane et al., 2022b; Song et al., 2024).

From the perspective of disease heterogeneity, this review synthesizes gut microbiome evidence across different DED phenotypes and proposes an analytical framework linking DED phenotypes, evidence stratification, and precision intervention. Based on study design and evidentiary directness, this review qualitatively stratifies gut microbiome evidence across DED phenotypes to clarify evidence strength and the boundaries of inference. Previous reviews have largely discussed the gut–eye axis in DED as a general concept, whereas fewer have systematically distinguished whether the strength and biological relevance of microbiome evidence differ across DED phenotypes. This review addresses this gap by integrating current evidence within a phenotype-stratified framework.

## 2 Phenotypic heterogeneity of dry eye disease and a framework for stratification analysis

DED heterogeneity extends beyond the conventional distinction between aqueous-deficient dry eye (ADDE) and evaporative dry eye (EDE), encompassing immune-inflammatory status, meibomian gland function, lacrimal secretory capacity, postoperative or environmental exposure, and symptom–sign concordance (Belmonte et al., 2017; Bron et al., 2017; Craig et al., 2017; Vehof et al., 2017; Ong et al., 2018; Ahn et al., 2022; Xiong et al., 2024). External factors, including ocular surgery, air pollution, low humidity, and prolonged digital screen exposure, may further contribute to clinically relevant DED phenotypes (Mehra and Galor, 2020; Ito and Takada, 2023; Vaughan et al., 2024; Cao et al., 2025; Ta et al., 2025; Teo et al., 2025; Valencia-Sandonís et al., 2025; Xu et al., 2026). This review does not seek to redefine DED classification. Rather, it uses Sjögren syndrome (SjS)-associated or other immune-mediated DED, MGD/EDE-dominant DED, non-SjS ADDE, postoperative or environment-related DED, and symptom–sign discordant DED as analytical units for comparing evidence strength and potential gut–ocular surface pathways across phenotypes.

Phenotype stratification directly influences the interpretation of gut microbiome studies in DED. If different DED phenotypes are pooled in the same analysis, phenotype-specific signals may be diluted, leading to overgeneralized mechanistic interpretations. The putative contribution of the gut–ocular surface axis may vary across phenotypes: immune-mediated DED is more closely linked to systemic immune remodeling; MGD/EDE-dominant DED may involve a metabolic–inflammatory background; non-SjS ADDE may be associated with aging-related lacrimal gland vulnerability; postoperative or environment-related DED may reflect differences in host inflammatory thresholds and tissue repair capacity; and symptom–sign discordant DED may involve neurosensory abnormalities and neuroimmune regulation (Belmonte et al., 2017; Bron et al., 2017; Vehof et al., 2017; Ong et al., 2018; Ahn et al., 2022; Xiong et al., 2024). Therefore, phenotype stratification is fundamental to microbiome study design and evidence integration.

## 3 Potential biological pathways linking the gut microbiome to ocular surface homeostasis

The gut–ocular surface axis may operate through multiple biological pathways, including immune-inflammatory, metabolic–barrier, and neuroimmune pathways. This section outlines these pathways as a mechanistic foundation for the subsequent phenotype-stratified synthesis of evidence.

### 3.1 Immune-inflammatory remodeling

Immune-inflammatory remodeling represents one of the major pathways through which the gut microbiota may influence DED. Gut dysbiosis can alter the host immune environment by disrupting the intestinal barrier, facilitating the translocation of microbiota-associated molecules and inflammatory signals into the circulation, and impairing mucosal immune tolerance (Watane et al., 2022b; Song et al., 2024). These systemic immune changes may further affect adaptive immune balance and pro-inflammatory cytokine networks, particularly the balance between regulatory T cells and pathogenic Th17 responses. As a result, gut dysbiosis may indirectly amplify inflammation in the lacrimal gland and ocular surface, thereby aggravating epithelial barrier disruption and tear film instability (Bron et al., 2017; Watane et al., 2022b).

In addition to immune cell regulation, gut microbial metabolites may modulate ocular surface inflammation. Short-chain fatty acids, particularly butyrate, are key microbial metabolites involved in mucosal immune regulation. Experimental evidence suggests that gut-derived butyrate or its precursor tributyrin can suppress desiccating stress-induced ocular surface inflammation and corneal barrier disruption, indicating that microbial metabolic function may be as important as microbial composition in maintaining ocular surface immune homeostasis (Schaefer et al., 2022a).

Inflammatory remodeling in DED should also be interpreted in relation to oxidative stress, epithelial stress responses, and cytokine-amplification loops at the ocular surface. Nutritional and microbiome-related factors may modulate these processes by influencing systemic low-grade inflammation, antioxidant capacity, lipid mediator balance, and immune polarization. Consistent with this view, the TFOS Lifestyle report has highlighted that nutrition may affect ocular surface health directly or indirectly through systemic metabolic and inflammatory conditions (Markoulli et al., 2023). Mediterranean diet components, including omega-3 fatty acids, polyphenols, dietary fiber, vitamins, and antioxidant-rich foods, may attenuate DED-related inflammation and oxidative stress through metabolic and microbiome-dependent pathways (Ahmadzadeh et al., 2026).

Thus, the immune-inflammatory pathway can be conceptualized as a sequential cascade: gut dysbiosis, intestinal barrier dysfunction, increased circulating microbial or inflammatory signals, impaired immune tolerance, Th17/Treg imbalance, lacrimal gland and ocular surface inflammation, oxidative stress and epithelial stress responses, epithelial barrier damage, and tear film instability. This pathway provides a mechanistic framework for interpreting microbiome-associated immune alterations in DED, but its clinical relevance is likely to be phenotype-dependent rather than applicable to all DED phenotypes.

### 3.2 Metabolic–barrier regulation and host susceptibility

Microbial metabolite production and intestinal barrier integrity are key components of the gut–ocular surface axis. The gut microbiota contributes to the generation of short-chain fatty acids, bile acids, amino acid derivatives, and other bioactive metabolites, which can regulate mucosal immune tolerance, epithelial barrier integrity, lipid metabolism, oxidative stress, and systemic low-grade inflammation (Labetoulle et al., 2024; Song et al., 2024). When microbial metabolism is disrupted or intestinal permeability increases, microbial products and inflammatory mediators may more readily enter the circulation, shifting the host toward a pro-inflammatory state and reducing tissue resilience. This pathway is particularly relevant to DED phenotypes in which the gut microbiota is unlikely to directly drive disease but may instead modulate host susceptibility. In some non-immune-dominant DED phenotypes, this pathway may manifest as modulation of metabolic susceptibility, inflammatory thresholds, and tissue repair capacity rather than as a direct driver of ocular surface pathology.

Dietary patterns further connect metabolism, inflammation, and microbiome function. Components of the Mediterranean diet, including omega-3 fatty acids, polyphenols, dietary fiber, vitamins, and antioxidant-rich foods, have been proposed as potential modulators of DED-related inflammation, oxidative stress, tear film lipid layer function, and gut microbial metabolism (Ahmadzadeh et al., 2026). These effects may be partly mediated by changes in gut microbial composition and metabolic outputs, providing an upstream dietary context for microbiome-mediated immune and metabolic regulation in DED (Perrone and D’Angelo, 2025; Ahmadzadeh et al., 2026). However, the causal chain linking diet, microbiome alterations, metabolic signaling, and ocular surface improvement remains incompletely established. Future studies should therefore integrate dietary records, microbiome profiling, metabolomics, and ocular surface endpoints rather than treating diet as an isolated lifestyle factor.

### 3.3 Neuroimmune regulation

In studies of the gut microbiota in DED, neuroimmune pathways remain largely hypothesis-generating. TFOS DEWS II has incorporated neurosensory abnormalities into the pathophysiological framework of DED, indicating that dry eye discomfort depends not only on visible ocular surface damage but also on inflammatory mediators, peripheral nerve sensitization, and altered sensory processing (Belmonte et al., 2017). The gut microbiota may influence host sensory regulation through the gut–brain axis, neuroimmune signaling, endocrine pathways, and microbial metabolites (Trujillo-Vargas et al., 2020; Watane et al., 2022b; Labetoulle et al., 2024). However, direct evidence for a “gut microbiota–neural sensitization–dry eye symptoms” pathway in patients with DED remains insufficient. Nevertheless, literature on chronic pain and gut–brain interactions suggests that microbial communities can influence neuroinflammation, pain sensitization, and central sensory processing (Pak et al., 2024; Lou et al., 2025). These mechanisms are theoretically relevant to symptom–sign discordant DED, but direct validation in DED populations is still lacking. Future studies should integrate pain phenotyping, psychological comorbidities, corneal nerve parameters, tear biomarkers, and microbial functional profiles. These pathways should not be interpreted as uniformly operating in all patients with DED. Rather, their clinical relevance is likely to depend on the dominant DED phenotype, systemic immune background, metabolic status, and neurosensory profile.

## 4 Phenotype-specific evidence and putative roles of the gut microbiome in DED

Building on the phenotypic framework outlined above, this section compares the strength, sources, and inferential limits of current evidence supporting the gut–ocular surface axis across distinct DED phenotypes.

**Table 1** summarizes the phenotype-stratified evidence landscape.

**Table 1:** Phenotype-stratified evidence for the gut–ocular surface axis in DED.

Phenotype	Main evidence	Putative mechanisms	Evidence appraisal	Research implication
SjS-associated/immune-mediated DED	Clinical microbiome studies; germ-free or antibiotic-treated models; patient-derived microbiota transplantation	Microbiota dysbiosis; impaired immune tolerance; Th17/Treg imbalance; amplified lacrimal gland–ocular surface inflammation	Relatively strongest evidence; human data remain mainly associative	Longitudinal cohorts, causal validation, and immunomodulatory microbiome-targeted interventions
MGD/EDE-dominant DED	MGD pathophysiology; local meibum/ocular surface microbiome studies; metabolic animal models	Lipid metabolic dysregulation, chronic low-grade inflammation, local microbiome changes, and host inflammatory susceptibility	Mainly indirect evidence; local microbiome changes do not equal gut-mediated systemic effects	Integrated assessment of gut microbiota, serum lipids, meibum composition, meibomian gland morphology, and tear film function
Non-SjS ADDE	Lacrimal gland aging studies; age-related or high-fructose models; tear film microbiome studies	Age-, diet-, and metabolic stress-related lacrimal gland vulnerability	Mainly animal and indirect evidence; human gut microbiome evidence is limited	Phenotype-stratified cohorts integrating microbiome and metabolomic analyses
Postoperative or environment-related DED	Postoperative dry eye studies; perioperative probiotic RCTs; environmental exposure studies	Altered inflammatory thresholds, barrier repair capacity, and ocular surface recovery	Early interventional signals for postoperative DED; environment-related DED remains inferential	Perioperative validation and prospective environmental exposure studies
Symptom–sign discordant DED	Symptom–sign discordance studies; ocular/chronic pain research; gut–brain axis inferences	Neurosensory abnormalities, pain sensitization, and gut–brain axis-related neuroimmune regulation	Hypothesis-generating evidence; direct validation in DED populations is lacking	Integrate pain phenotypes, corneal nerve parameters, tear inflammation, and microbiome function

ADDE, aqueous-deficient dry eye; DED, dry eye disease; EDE, evaporative dry eye; MGD, meibomian gland dysfunction; RCT, randomized controlled trial; SjS, Sjögren syndrome; Th17, T helper 17 cells; Treg, regulatory T cells.

### 4.1 Immune-mediated DED: SjS-associated DED as a representative phenotype

Among the various DED phenotypes, SjS-associated DED currently represents the most informative clinical model for investigating the gut–ocular surface immune axis. Compared with non-stratified DED populations, SjS-associated DED has a more clearly defined systemic autoimmune background, and ocular surface damage in this phenotype is not limited to aqueous tear deficiency but also involves immunopathological processes, including lacrimal gland inflammation, immune cell infiltration, and impairment of the ocular surface barrier (Saram et al., 2025; Soyfoo et al., 2025). Therefore, this phenotype provides a more appropriate framework for examining whether gut microbiota dysbiosis is linked to systemic immune remodeling and lacrimal gland–ocular surface inflammatory responses.

Existing clinical studies have shown that patients with SjS or immune-mediated dry eye exhibit altered gut microbial composition, and that some microbial changes are associated with dry eye severity or immune-related clinical indicators (Mendez et al., 2020; Moon et al., 2020a; Yao et al., 2022; Goodman et al., 2023; Yuan et al., 2025). Studies by Moon et al. and Goodman et al. suggest that gut microbial alterations can be detected in clinically defined SjS or immune-mediated dry eye populations and may differ across immune-mediated dry eye phenotypes (Moon et al., 2020a; Goodman et al., 2023). Mendez et al. also reported gut microbial dysbiosis in patients with SjS (Mendez et al., 2020). Furthermore, alterations in microbial diversity, community structure, and predicted functional profiles in patients with primary SjS suggest that microbiota abnormalities may not merely reflect background variation, but may also be involved in disease-related immune remodeling (Yao et al., 2022; Yuan et al., 2025). Nevertheless, most available human studies are cross-sectional or case-control in design and therefore primarily demonstrate associations between microbiota alterations and SjS-related dry eye; they cannot yet establish temporality or causality.

de Paiva et al. analyzed the conjunctival, tongue, and fecal microbiomes of patients with SjS and further integrated these human findings with an antibiotic-induced gut dysbiosis model under desiccating stress. They found that SjS disease severity was associated with altered mucosal microbiome diversity, and that disruption of the gut microbiota could exacerbate ocular surface inflammation and barrier damage (De Paiva et al., 2016). The value of this type of study lies not merely in describing microbial differences in patients, but in linking mucosal and fecal microbiome abnormalities in humans with ocular surface pathology in animal models. This provides a more coherent line of evidence supporting the “microbiome imbalance–immune inflammation–ocular surface damage” axis in SjS-associated dry eye.

Animal and mechanistic studies further strengthen the biological plausibility of this association. Germ-free mice can develop SjS-like inflammation involving the lacrimal glands, cornea, and conjunctiva, accompanied by corneal barrier disruption, goblet cell loss, and inflammatory changes, suggesting that commensal microorganisms may contribute to the maintenance of lacrimal gland–ocular surface immune homeostasis (Wang et al., 2018). Patient-derived microbiota transplantation studies provide more direct functional evidence. After gut microbiota from patients with SjS was transplanted into mice, recipient animals showed reduced Treg cells in lymphoid organs and developed more severe corneal barrier disruption under desiccating stress conditions (Schaefer et al., 2022b). In addition, a high-fat diet-induced SjS model showed that exacerbation of ocular surface and glandular lesions was accompanied by reduced gut microbial richness and altered community structure, and these microbial changes correlated with ocular surface, glandular, and serological parameters (Zhang et al., 2022). Microbiota transfer studies further suggest that dysbiotic gut microbiota from patients with SjS or from autoimmune dry eye mouse models may transfer susceptibility to ocular surface inflammation to normal mice; however, these findings have so far been reported mainly as conference abstracts and require confirmation in full peer-reviewed studies (Kim et al., 2024).

Therefore, SjS-associated dry eye and other immune-mediated forms of DED currently represent the phenotypes with the strongest and most convergent evidence for involvement of the gut–ocular surface axis. This evidence comes from clinical association studies, human mucosal and fecal microbiome analyses, germ-free animal models, antibiotic-induced dysbiosis models, and patient-derived microbiota transplantation experiments. Nevertheless, current human evidence remains largely correlational, and findings from animal and transplantation studies cannot substitute for longitudinal patient follow-up or interventional validation. SjS-associated dry eye should therefore be prioritized in future studies aimed at causal validation and microbiome-targeted intervention; however, evidence from this phenotype should not be generalized to suggest that gut microbiota dysbiosis drives the onset or progression of all DED phenotypes (Watane et al., 2022a).

### 4.2 MGD/EDE-dominant DED and non-SjS ADDE: indirect evidence and metabolic susceptibility

MGD/EDE-dominant DED and non-SjS ADDE differ in their primary ocular pathologies: the former is driven mainly by meibomian gland lipid abnormalities, increased tear evaporation, and local inflammation, whereas the latter is characterized by reduced lacrimal gland secretory reserve and impaired maintenance of ocular surface homeostasis. However, for both phenotypes, current evidence implicating the gut microbiota remains largely indirect and mechanistic rather than directly causal. Accordingly, this review considers them within the same evidentiary tier and focuses on whether the gut microbiota may modulate these phenotypes through effects on host metabolic susceptibility, inflammatory thresholds, and ocular surface resilience and repair capacity (Ahn et al., 2022; Donthineni et al., 2023; Rasaruck et al., 2023; Beatty et al., 2024; Naqvi et al., 2024; Galor and Margolis, 2025; Pei and Li, 2025; Zheng et al., 2025).

MGD/EDE-dominant DED is a common clinical phenotype of DED, with core pathological features including abnormal meibomian lipid secretion, ductal keratinization, increased tear evaporation, and local inflammatory responses (Beatty et al., 2024). However, direct clinical evidence demonstrating the involvement of the gut microbiota in the onset and progression of MGD/EDE remains lacking. Current links are mainly based on indirect evidence related to systemic metabolism, chronic low-grade inflammation, and mucosal homeostasis (Zheng et al., 2025). From a pathophysiological perspective, if the gut microbiota contributes to MGD/EDE, its role is more likely to involve modulation of lipid metabolism, systemic low-grade inflammatory status, and host inflammatory susceptibility, rather than direct induction of local ocular surface pathology (Beatty et al., 2024; Galor and Margolis, 2025). Metabolic animal models further support the notion that systemic lipid metabolic disorders can affect meibomian gland structure and function. A high-fat diet can induce meibomian gland inflammation and dysfunction in mice, accompanied by reduced PPAR-γ expression and activation of MAPK and NF-κB inflammatory signaling pathways; meanwhile, obese mice with dyslipidemia may develop meibomian gland hypertrophy, altered meibum lipid composition, and abnormal tear secretion (Osae et al., 2020; Bu et al., 2021). Therefore, the current understanding of the gut–ocular surface axis in MGD/EDE is better positioned as an indirect modulation of the local eyelid margin microenvironment by systemic metabolic and inflammatory contexts, rather than as an established direct causal pathway (Galor and Margolis, 2025; Zheng et al., 2025).

Meanwhile, alterations in the local microbial composition of the eyelid margin, meibum, or ocular surface in MGD/EDE should not be equated with gut microbiota-mediated systemic immune or metabolic regulation. Existing studies have shown that patients with MGD or DED may exhibit changes in the microbiota of meibum, the ocular surface, or other local ocular microenvironments (Yoon et al., 2021; Rasaruck et al., 2023; Naqvi et al., 2024). Rasaruck et al., for example, sequenced the meibum microbiota of patients with MGD and analyzed its relationship with tear cytokine levels, suggesting that the local microbiome may contribute to the inflammatory microenvironment of the eyelid margin (Rasaruck et al., 2023). However, these findings are derived mainly from local samples, including meibum, the eyelid margin, or the ocular surface, and therefore remain observations at the level of the local ocular microbiome. They cannot be directly extrapolated to gut microbiota-mediated systemic effects. Future studies should therefore integrate assessments of the gut microbiota, lipid metabolism, systemic inflammation, meibum composition, meibomian gland morphology, and tear film function (Ozkan et al., 2023; Rasaruck et al., 2023; Beatty et al., 2024; Naqvi et al., 2024; Galor and Margolis, 2025).

Unlike SjS-associated autoimmune lacrimal gland damage, non-SjS ADDE lacks a clearly defined systemic autoimmune background and is more likely to be related to age-related lacrimal gland functional decline, medication exposure, abnormal neural regulation, and altered host metabolic status (Donthineni et al., 2023; Pei and Li, 2025). Therefore, in non-SjS ADDE, the relevance of the gut microbiota is better interpreted within the framework of “aging/metabolic milieu–host homeostatic vulnerability–lacrimal gland dysfunction”.

At present, evidence suggesting a possible role of the gut microbiota in non-SjS ADDE comes mainly from animal and metabolic studies. For example, age-related gut microbiota dysbiosis has been associated with dry eye severity in mice, and long-term high-fructose intake can promote lacrimal gland dysfunction by inducing gut dysbiosis (Yoon et al., 2021; Qi et al., 2023). In addition, tear film microbiome studies have shown that both Sjögren and non-Sjögren aqueous-deficient dry eye may be accompanied by alterations in the tear film bacterial microbiome, suggesting that non-SjS ADDE may also involve local ocular surface microbial abnormalities (Pal et al., 2023). Together, these findings suggest that diet, aging, the metabolic milieu, and local ocular surface microbiome alterations may jointly affect lacrimal gland–ocular surface homeostasis. However, the strength of this evidence remains lower than that of the clinical association studies and microbiota transplantation-based mechanistic studies available for SjS-associated dry eye (Yoon et al., 2021; Pal et al., 2023; Qi et al., 2023).

Overall, MGD/EDE-dominant DED and non-SjS ADDE are currently supported mainly by indirect evidence from metabolic studies, local microbiome research, and animal mechanistic models, and direct causal evidence remains lacking. Future studies should avoid simply combining these phenotypes with immune-mediated dry eye and should instead clarify the true role of the gut microbiota within each phenotype.

### 4.3 Postoperative, environment-related, and symptom–sign discordant DED: host susceptibility and neuroimmune hypotheses

Postoperative, environment-related, and symptom–sign discordant DED differ from SjS-associated DED and MGD/EDE-dominant DED in both clinical context and evidentiary basis. They are grouped here not because they share a single pathophysiological mechanism, but because current microbiome-related evidence for these phenotypes is largely indirect, preliminary, or hypothesis-generating. A shared feature of these phenotypes is the scarcity of direct evidence implicating the gut microbiota. Current interpretations are therefore based mainly on indirect links among external stimuli, host susceptibility, tissue repair capacity, and neuroimmune regulation. These phenotypes should not be interpreted as disease models directly driven by the gut microbiota. Rather, they are better regarded as exploratory settings for investigating “external stimuli–host response–ocular surface recovery” and “neuroimmune regulation–symptom amplification” pathways.

Postoperative and environment-related DED have relatively well-defined external triggers, including corneal refractive surgery, cataract surgery, prolonged digital screen exposure, low-humidity environments, and air pollution. These factors can disrupt ocular surface homeostasis through tear film instability, corneal nerve injury, increased tear evaporation, and ocular surface inflammation (Mehra and Galor, 2020; Ito and Takada, 2023; Vaughan et al., 2024; Cao et al., 2025; Teo et al., 2025; Valencia-Sandonís et al., 2025; Xu et al., 2026). In these phenotypes, the gut microbiota is more likely to act as a modulatory factor influencing host inflammatory thresholds, barrier repair capacity, and interindividual differences in postoperative recovery, rather than as a direct etiological driver. The most direct evidence comes from perioperative microbiome intervention studies. A randomized, double-blind, placebo-controlled trial showed that patients undergoing refractive surgery who received Bifidobacterium bifidum BB00 at 2 × 10^9^ CFU/day for 30 consecutive days had lower postoperative dry eye incidence and improved OSDI scores, FBUT, and Schirmer I test results compared with the placebo group. This study also assessed gut microbiota changes using 16S rRNA sequencing (Cheng et al., 2026). These findings suggest that perioperative microbiome intervention may influence postoperative ocular surface recovery; however, the evidence remains preliminary and requires validation in additional independent randomized controlled trials.

In contrast, evidence linking the human gut microbiota to environment-related DED remains more limited. Factors such as low humidity, air pollution, and prolonged digital screen exposure primarily affect ocular surface homeostasis through local ocular surface stress, increased tear evaporation, inflammatory responses, and altered blinking behavior (Mehra and Galor, 2020; Ito and Takada, 2023; Vaughan et al., 2024; Cao et al., 2025; Teo et al., 2025; Valencia-Sandonís et al., 2025; Xu et al., 2026). To date, prospective population-based studies directly demonstrating that gut microbiota alterations contribute to the onset or progression of environment-related DED are still lacking. Therefore, in this phenotype, the gut–ocular surface axis is better understood as a potential background factor that modulates host responses to environmental stress and ocular surface recovery capacity, rather than as an established causal pathway.

Symptom–sign discordant DED represents another phenotype with relatively weak evidence but considerable value for mechanistic exploration. Its defining feature is the presence of pronounced subjective symptoms despite relatively mild or incongruent objective ocular surface damage. Neuropathic abnormalities, impaired pain modulation, and systemic pain susceptibility may contribute to symptom generation, rather than symptoms being determined solely by local ocular surface damage (Matar et al., 2026). Clinical studies indicate that patients with prominent symptoms but mild signs more frequently present with chronic pain tendency, depression, or non-ocular pain comorbidities, suggesting that this phenotype may be linked to systemic pain modulation and neurosensory abnormalities (Vehof et al., 2017; Ong et al., 2018; Xiong et al., 2024). Further studies have shown that dry eye-related neuropathic ocular pain can coexist with various chronic pain syndromes, supporting the possibility that some patients with DED have systemic pain susceptibility or a background of central sensitization (Galor et al., 2016).

Baseline studies of the gut microbiota in symptom–sign discordant DED remain limited, and current inferences are derived mainly from animal models or from studies of chronic pain, neuroinflammation, and the gut–brain axis in other disease contexts. The gut microbiota may influence pain sensitization and symptom perception through neural networks, inflammatory mediators, hormonal axes, and microbial metabolic signals, thereby exacerbating subjective discomfort in patients with pronounced symptoms but relatively mild signs (Pak et al., 2024; Tîrziu et al., 2024; Lou et al., 2025). However, this mechanism has not yet been directly validated in DED. At present, this phenotype should therefore be regarded as a hypothesis-generating model for exploring the relationships among the gut microbiota, neuroimmune regulation, and pain sensitization, rather than as an independent subtype with well-established evidence for the gut–ocular surface axis.

Postoperative, environment-related, and symptom–sign discordant DED are currently supported mainly by early interventional signals, external exposure studies, and neuroimmune inferences, with a lower level of evidence than SjS-associated dry eye and metabolically susceptible phenotypes. Therefore, these phenotypes should not be regarded as models directly driven by the gut microbiota, but are better positioned as exploratory models for validating “host susceptibility modulation” and “neuroimmune symptom amplification”.

## 5 Advances in microbiome-targeted interventions and the role of phenotypic stratification in treatment

### 5.1 Probiotics, prebiotics, synbiotics, and postbiotics: preliminary clinical signals, mechanistic evidence, and heterogeneous efficacy

Probiotics, prebiotics, synbiotics, and postbiotics are currently the most commonly investigated microbiome-targeted strategies in DED. Unlike ecological-level microbiome reconstruction approaches such as fecal microbiota transplantation (FMT), these interventions usually aim to modulate host immunity, metabolism, and barrier function by supplementing specific microbial strains, providing substrates that promote the growth of beneficial bacteria, or applying functional products derived from microorganisms. Existing studies suggest that these interventions may improve dry eye symptoms, tear film stability, tear secretion, and inflammation-related markers in some patients with DED. However, their efficacy remains inconsistent, and substantial heterogeneity exists across studies in terms of strain composition, formulation, route of administration, treatment duration, study population, and outcome measures (Chisari et al., 2017; Tavakoli et al., 2022; Heydari et al., 2024; Yen et al., 2025; Tavakoli et al., 2026). Therefore, at the current stage, probiotics or synbiotics should not be regarded as universal therapeutic options for DED.

In human studies, early clinical evidence suggested that synbiotic supplementation containing Bifidobacterium and fructo-oligosaccharides may improve ocular surface damage and tear film-related parameters in some patients with dry eye (Chisari et al., 2017). A double-blind randomized controlled trial by Tavakoli et al. further showed that oral probiotics and prebiotics may improve DED symptoms and selected ocular surface parameters, indicating that gut microbiota modulation has potential therapeutic relevance (Tavakoli et al., 2022). However, findings across clinical studies are not fully consistent. Subsequent studies evaluating ocular and systemic inflammatory endpoints suggest that improvements in symptoms, ocular surface signs, and inflammatory markers may not occur in parallel, indicating that the clinical benefits of microbiome-targeted interventions cannot be judged by a single endpoint alone (Tavakoli et al., 2026). Furthermore, a triple-masked, randomized, placebo-controlled trial by Heydari et al. showed that a topical postbiotic formulation containing Latilactobacillus sakei lysate improved DED signs and symptoms and suppressed ocular surface inflammatory responses, whereas oral L. sakei capsules alone showed no clear efficacy (Heydari et al., 2024). These findings suggest that the effects of microbiome-targeted interventions depend not only on the use of probiotic organisms, but also on strain specificity, formulation, route of administration, site of action, and patient phenotype.

Animal and cellular studies have provided clearer mechanistic insights into these interventions. In an experimental mouse model of dry eye, Moon et al. found that treatment with IRT5 probiotics partially alleviated dry eye manifestations and increased tear secretion, and that these improvements were accompanied by changes in the gut microbiome. These findings suggest that the gut microbiota may influence lacrimal gland function through mechanisms beyond inflammatory regulation (Moon et al., 2020b). Choi et al. further demonstrated in an autoimmune dry eye mouse model that IRT5 probiotics altered the expression of proteins involved in lacrimal gland immune regulation and improved ocular surface damage and tear secretion, suggesting that probiotic intervention may exert its effects by reshaping the immune microenvironment of the lacrimal gland (Choi et al., 2020). In addition to live bacterial interventions, probiotic-derived exosomes, postbiotics, and other microbial-derived functional products have shown anti-inflammatory and ocular surface-protective potential, potentially representing emerging directions for microbiome-targeted interventions (Heydari et al., 2024; Lee et al., 2024). However, these mechanistic findings require further validation in human DED studies with well-defined phenotypes. Although animal and cellular studies are useful for elucidating immune, oxidative stress-related, and metabolic pathways, differences in model background, induction methods, and intervention doses limit the direct extrapolation of these findings to all patients with DED.

Beyond IRT5, strain-specific evidence is also emerging. Chang et al. showed that Streptococcus thermophilus iHA318 improved ocular surface damage, tear secretion, and tear film stability *in vitro* and in a UVB-induced mouse model of dry eye by reducing oxidative stress and inflammatory responses (Chang et al., 2024). Subsequently, a randomized clinical trial by Yen et al. suggested that oral administration of S. thermophilus iHA318 improved some dry eye symptoms and tear-related parameters in humans (Yen et al., 2025). This continuum from animal mechanistic evidence to human clinical signals has greater translational significance than animal studies alone; nevertheless, further validation in independent cohorts and across different DED phenotypes is still required.

Recent animal and mechanistic studies further suggest that microbiome-targeted interventions may be phenotype-dependent and strain-specific. In a type 2 diabetes-associated dry eye-like mouse model, Dai et al. found that probiotic and prebiotic interventions improved dry eye-like manifestations and related pathological changes, accompanied by gut microbiota remodeling and reduced bile acid metabolites. These findings suggest that, in the context of metabolic abnormalities, microbiome-targeted interventions may act through a “gut microbiota–bile acid metabolism–ocular surface homeostasis” pathway (Dai et al., 2025). In addition, Lactobacillus fermentum HY7302 has been shown to alleviate ocular surface inflammation by downregulating inflammatory and tissue damage-related factors, including IL-1β, IL-6, IL-8, and MMP-9. Its derived exosomes or bacterial extracellular vesicles may also improve ocular surface inflammation-related dry eye through extracellular vesicle-mediated gut–ocular surface communication, supporting postbiotics and microbial extracellular vesicles as novel intervention modalities that do not rely on live bacterial colonization (Lee et al., 2023; Lee et al., 2024). These studies indicate that microbiome-targeted interventions should not be understood simply as “probiotic supplementation.” Instead, they should be further differentiated according to microbial strain, functional product, delivery form, host metabolic background, and DED phenotype.

Among these strategies, clinical evidence is currently strongest for selected probiotic or synbiotic formulations, whereas postbiotics, extracellular vesicles, and strain-derived products remain more mechanistically promising than clinically established. Therefore, probiotics, prebiotics, synbiotics, and postbiotics are currently better positioned as candidate strategies for phenotype-directed intervention studies rather than as universal therapeutic regimens for all patients with DED. SjS-associated and other immune-mediated forms of DED may serve as priority populations for validating immunomodulatory microbiome-targeted interventions. MGD/EDE-dominant DED and non-SjS ADDE are better interpreted from the perspectives of metabolic susceptibility and lacrimal gland functional reserve. Postoperative DED has shown early signals for perioperative microbiome intervention, whereas environment-related and symptom–sign discordant DED still lack direct interventional evidence.

### 5.2 Fecal microbiota transplantation and emerging microbiome-based therapies: proof of concept rather than routine clinical application

Fecal microbiota transplantation (FMT) differs fundamentally from interventions based on single or limited components, such as probiotics, prebiotics, or postbiotics. Rather than supplementing a specific beneficial strain, FMT aims to reconstruct the recipient gut microbial ecosystem by transferring a complex donor-derived microbiota. Accordingly, FMT is better conceptualized as an ecological-level microbiome reconstruction strategy. In DED, the most theoretically relevant candidates for this approach are not unselected patients, but those with SjS-associated or other immune-mediated forms of DED, given their systemic immune abnormalities, disruption of mucosal immune tolerance, Treg/Th17 imbalance, and lacrimal gland–ocular surface inflammation.

Currently, direct clinical evidence for FMT in DED remains very limited. Watane et al. conducted a small-sample, open-label study involving patients with immune-mediated dry eye. Under the study conditions, FMT appeared to be feasible and safe; some patients reported improvements in subjective dry eye symptoms at 3 months, and the gut microbiota profiles of some recipients shifted toward those of the donors during follow-up (Watane et al., 2022a). The importance of this study lies in its role as the first human exploration of FMT as a microbiome-based intervention for immune-mediated dry eye, providing early clinical evidence that gut microbiota remodeling may influence ocular surface symptoms. However, its limitations are substantial. The study had a small sample size and lacked randomization, masking, and placebo control; its outcomes included subjective symptom improvement; the follow-up period was limited; and it could not determine whether symptom changes resulted from microbiota remodeling, placebo effects, natural disease fluctuation, or concomitant treatments. Watane et al. also noted that the optimal route and protocol for FMT administration remain undetermined (Watane et al., 2022a).

The biological rationale for FMT is derived mainly from mechanistic studies of immune-mediated dry eye. Germ-free animal models, antibiotic-induced dysbiosis models, and patient-derived microbiota transplantation experiments all suggest that the gut microbiota can influence lacrimal gland–ocular surface immune homeostasis. Schaefer et al. transplanted gut microbiota from patients with SjS into mice and observed reduced Treg cells in eye-associated lymphoid tissues, together with more severe corneal barrier disruption under desiccating stress conditions. These findings suggest that SjS-associated microbiota abnormalities may affect ocular surface homeostasis through immune remodeling (Schaefer et al., 2022b). Accordingly, existing animal and transplantation studies support the use of FMT primarily as a causal validation tool or mechanistic probe, rather than as an established therapeutic modality.

From a clinical translation perspective, FMT faces greater safety and standardization requirements in DED than probiotics or postbiotics. FMT involves multiple variables, including donor screening, risk of pathogen transmission, preparation procedures, route of administration, dosage, repeated dosing, recipient immune status, and long-term follow-up. Existing regulatory and guideline evidence has focused primarily on recurrent Clostridioides difficile infection rather than ocular surface diseases. The 2024 AGA guidelines mainly address gastrointestinal indications, such as recurrent C. difficile infection, and therefore cannot be extrapolated as a therapeutic basis for DED (Peery et al., 2024). Therefore, when discussing FMT in the context of DED, it should not be presented as a clinically scalable treatment in the near term, but rather as an investigational intervention that requires strict regulatory oversight.

At present, the role of FMT in DED is best positioned as a proof-of-concept and mechanistic exploration tool for immune-mediated dry eye, rather than as a routine treatment option. For MGD/EDE-dominant DED, non-SjS ADDE, environment-related DED, and symptom–sign discordant DED, direct evidence supporting FMT is currently lacking; therefore, FMT should not be considered a priority intervention for these phenotypes.

### 5.3 Design principles for phenotype-directed microbiome intervention studies

Translational research on microbiome-targeted interventions for DED should move beyond simply asking whether a given intervention is effective and instead focus on phenotype-directed studies that test predefined mechanistic hypotheses. Future trials should avoid enrolling heterogeneous DED populations with different pathological backgrounds without stratification. The target phenotype, core mechanistic hypothesis, and intervention objective should be clearly defined before enrollment. For immune-mediated dry eye, studies should prioritize immune-inflammatory responses and lacrimal gland–ocular surface barrier endpoints. For metabolically susceptible phenotypes, lipid metabolism, bile acids, and short-chain fatty acids should be integrated into the assessment. For postoperative DED, ocular surface repair, corneal nerve recovery, and the incidence of postoperative dry eye should be key outcomes.

Diet-derived bioactive compounds, including Mediterranean diet-related polyphenols, may represent potential adjunctive strategies related to microbiome modulation. Evidence from non-DED immune-mediated neurological conditions suggests that polyphenols such as ellagic acid may influence inflammation-related symptoms and neuroimmune status; however, their relevance to DED-specific inflammatory or neurosensory pathways remains to be established. Future DED studies should therefore define target phenotypes, dietary exposure, microbial and metabolic endpoints, inflammatory biomarkers, neurosensory outcomes, and ocular surface parameters before their role as mechanism-directed interventions can be established (Karegar et al., 2025).

Study designs should further standardize microbial strains or FMT protocols, dosage, route of administration, treatment duration, and concomitant therapy, while concurrently recording OSDI, FBUT, Schirmer test results, corneal staining, tear inflammatory factors, gut microbiota composition, functional metabolites, and safety outcomes. Only when the target phenotype, intervention mechanism, and outcome measures are aligned can microbiome-targeted interventions progress from exploratory adjunctive strategies to precision adjunctive therapy.

Representative microbiome-targeted interventions are summarized in **Table 2**.

**Table 2:** Representative microbiome-targeted interventions in DED.

Intervention/category	Evidence source	Key outcomes	Mechanistic implications	Evidence level	Main limitations/positioning
Synbiotics, probiotics, and prebiotics	Early clinical study and RCTs in DES/DED patients (Chisari et al.; Tavakoli et al.)	Improved OSDI and selected tear/ocular surface parameters in some studies; signs and inflammatory endpoints were not consistently synchronized	Possible modulation of gut microbiota and systemic/ocular inflammatory status; mechanistic readouts remain limited	Preliminary human clinical evidence	Small cohorts, heterogeneous formulations, and insufficient phenotype stratification
Topical postbiotic/oral probiotic	Triple-masked RCT in DED patients (Heydari et al.)	Topical L. sakei lysate improved OSDI, TBUT, Schirmer I, and inflammatory outcomes; oral capsules alone showed unclear efficacy	Reduced tear inflammatory cytokines; highlights importance of formulation and route of administration	Single human RCT	Needs replication; efficacy may depend on delivery route and target site
IRT5 composite probiotics	Autoimmune and environmentally induced dry eye mouse models (Kim, Moon, Choi et al.)	Reduced ocular surface staining and increased tear secretion in autoimmune models; response varied by model	Increased Treg-related regulation, reduced proinflammatory immune responses, and altered lacrimal gland immune proteins	Animal mechanistic evidence	Model-dependent efficacy; limited direct extrapolation to humans
S. thermophilus iHA318	UVB-induced dry eye mice plus human RCT in DES volunteers (Chang et al.; Yen et al.)	Improved tear volume, TBUT, corneal staining, and selected symptoms/tear parameters	Antioxidant and anti-inflammatory effects; possible NLRP3-related modulation	Animal-to-human translational signal	Requires independent validation and phenotype-stratified trials
Microbiome intervention in metabolic context	Type 2 diabetes-associated dry eye-like mouse model (Dai et al.)	Improved dry eye-like manifestations and reduced corneal/lacrimal gland damage	Gut microbiota remodeling, reduced bile acid metabolites, and TLR4/NF-κB pathway suppression	Metabolism-related animal evidence	Supports metabolic-susceptibility hypothesis; human evidence is lacking
Postbiotics/microbial extracellular vesicles	BAC-induced dry eye mice and conjunctival cell models (Lee et al.)	Improved corneal staining, TBUT, tear secretion, and cellular injury markers	Downregulated IL-1β, IL-6, IL-8, MMP-9; modulated apoptosis and tight-junction pathways	Preclinical mechanistic evidence	Human efficacy and safety remain unvalidated
Perioperative probiotic	Double-blind RCT in FS-LASIK patients (Cheng et al.)	Reduced postoperative DED incidence and improved OSDI, FBUT, and Schirmer I	Partial reversal of postoperative gut dysbiosis by 16S rRNA sequencing	Human double-blind RCT evidence	Most applicable to perioperative settings; needs external validation
FMT	Open-label study in immune-mediated DED/SjS-associated disease (Watane et al.)	Feasible and generally safe under study conditions; some patients reported symptom improvement at 3 months	Recipient microbiota shifted toward donor profiles; immune profiles may relate to symptoms	Early proof-of-concept evidence	Small, open-label, no placebo control; investigational rather than routine therapy
Diet-related microbiome modulation	Narrative/mechanistic evidence on Mediterranean diet components (Ahmadzadeh et al.)	Possible improvements in TBUT, Schirmer test, and OSDI, but findings are inconsistent	Anti-inflammatory/antioxidant effects, lipid-layer support, and microbiota-metabolic modulation	Indirect mechanistic evidence	Overall dietary-pattern evidence remains weak; needs controlled phenotype-stratified studies

BAC, benzalkonium chloride; DED, dry eye disease; DES, dry eye syndrome; FMT, fecal microbiota transplantation; IL, interleukin; MMP-9, matrix metalloproteinase-9; OSDI, Ocular Surface Disease Index; RCT, randomized controlled trial; SjS, Sjögren syndrome; T2DM, type 2 diabetes mellitus; TBUT, tear breakup time; UVB, ultraviolet B,NF-κB, nuclear factor kappa B; TLR4, Toll-like receptor 4.

## 6 Current limitations and future research directions

### 6.1 From correlation to causation: the need for longitudinal cohorts and mechanistic validation

Most existing studies on DED and the gut microbiota are small-sample cross-sectional, case-control, or early exploratory interventional studies. As a result, most findings can only demonstrate associations between microbiota alterations and DED phenotypes, and it remains difficult to establish temporality or causal direction. Although Mendelian randomization studies have suggested that certain gut microbial taxa may be associated with the risk of SjS, these results are influenced by data sources, phenotype definitions, and instrumental variable selection, and therefore cannot substitute for direct mechanistic evidence (Zhao et al., 2025). Future studies should integrate prospective cohorts, germ-free or antibiotic-treated animal models, patient-derived microbiota transplantation, and targeted metabolite supplementation experiments to validate the causal chain linking “microbiota dysbiosis–intestinal barrier disruption–immune/metabolic remodeling–lacrimal gland and ocular surface damage”.

### 6.2 From mixed DED populations to phenotype-stratified microbiome research

DED is highly heterogeneous, with underlying mechanisms, patterns of ocular surface damage, and treatment responses varying across phenotypes (Craig et al., 2017; Yu et al., 2022; Chen et al., 2024). If SjS-associated DED, MGD/EDE-dominant DED, non-SjS ADDE, postoperative or environment-related DED, and symptom–sign discordant DED are analyzed together without stratification, true microbiome signals within specific phenotypes may be diluted, leading to overly generalized mechanistic interpretations (Labetoulle et al., 2024; Song et al., 2024). In addition, factors such as age, sex, diet, medication use, metabolic disease, psychological comorbidities, visual behavior, and environmental exposure may influence both gut microbiota composition and DED severity.

Future studies should predefine stratification variables within a unified diagnostic framework, including SjS status, MGD severity, tear secretion level, postoperative or environmental exposure history, symptom–sign concordance, and pain phenotype. This would shift the research focus from “whether microbiome differences exist in DED” to “which DED phenotypes are most likely to be influenced by the gut microbiota, and through which mechanisms”.

### 6.3 From microbial composition profiling to functional multi-omics and mechanism-directed intervention trials

Most current studies remain limited to 16S rRNA sequencing, α/β diversity analyses, and descriptions of differentially abundant bacterial genera. Functional evidence consistently linking microbiome alterations to metabolites, host immunity, tear inflammatory factors, and ocular surface clinical endpoints remains insufficient. Differences in sample collection and storage, DNA extraction, sequencing platforms, taxonomic annotation, functional prediction, and statistical workflows may also affect the reproducibility of results. Future studies should follow human microbiome reporting guidelines, such as STORMS, and provide detailed information on participant inclusion criteria, dietary and medication exposures, sample handling, sequencing platforms, analytical workflows, confounder control, and statistical methods. They should also integrate metagenomics, metabolomics, immunophenotyping, tear biomarkers, corneal nerve parameters, and conventional ocular surface indicators to validate the mechanistic link among “microbiota function–metabolic signaling–host response–ocular surface phenotype” (Mirzayi et al., 2021; Labetoulle et al., 2024; Song et al., 2024).

Microbiome-targeted intervention studies should also shift from general supplementation strategies toward phenotype-directed mechanistic validation. Although probiotics, prebiotics, synbiotics, postbiotics, and FMT have shown exploratory value, their efficacy stability, target populations, long-term safety, and reproducibility remain unclear (Chisari et al., 2017; Kim et al., 2017; Heydari et al., 2024; Berzack and Galor, 2025; Yen et al., 2025; Tavakoli et al., 2026). Future studies should combine clinical outcomes with analyses of gut microbiota function, metabolites, and host immune markers within a framework of phenotypic stratification and standardized reporting, thereby improving the interpretability and reproducibility of findings. For ecosystem-level interventions such as FMT, higher standards for safety, standardization, and long-term follow-up are required, and relevant indications should not be prematurely extrapolated to routine DED treatment (Peery et al., 2024). Only through phenotypic stratification, functional validation, and standardized reporting can research on the gut–ocular surface axis in DED move from exploratory associations toward precision adjunctive therapeutic translation.

## 7 Conclusions

In summary, the gut microbiota may influence ocular surface homeostasis through immune-inflammatory, metabolic-barrier, and neuroimmune pathways, but its effects are unlikely to be uniform across DED phenotypes. Current evidence is most robust for SjS-associated and other immune-mediated forms of DED, whereas evidence for MGD/EDE-dominant DED, non-SjS ADDE, postoperative or environment-related DED, and symptom–sign discordant DED remains largely indirect, preliminary, or hypothesis-generating. Future studies should place disease heterogeneity at the center of research design and integrate phenotype-stratified cohorts, causal validation, functional multi-omics, and mechanism-directed trials. This approach will be essential for clarifying the role of the gut–ocular surface axis in distinct DED phenotypes and for advancing microbiome-targeted interventions from exploratory concepts toward precision adjunctive therapy. The central challenge is therefore to move beyond identifying gut microbial differences in DED and to determine which phenotypes are biologically and clinically actionable through microbiome-targeted strategies.
