# Supplementary material for: Gut–ocular surface axis in dry eye disease: phenotype-specific mechanisms, evidence, and microbiome-targeted interventions
Source: Front Cell Infect Microbiol. 2026 Jul 6;16:1873050. doi: 10.3389/fcimb.2026.1873050 (PMC13381242; doi:10.3389/fcimb.2026.1873050)
Supplement: Supplementary file 1 [file Table1.docx]

Supplementary Material

## **Table 2. Representative microbiome-targeted interventions for DED**

| **Intervention Type** | **Research Subject/Model** | **Study Design** | **Primary Outcome** | **Mechanism Indicators** | **Level of Evidence** | **Major Limitations** |
| --- | --- | --- | --- | --- | --- | --- |
| Synbiotics | DES/DED patients, n=40 (Chisari et al., 2017) | Early clinical study; oral fructo-oligosaccharides plus Bifidobacterium strains | Improved Schirmer I/II and BUT; reduced conjunctival culture positivity | Reduced aerobic/anaerobic isolates; no gut microbiome sequencing | Early clinical evidence | Small sample; no phenotype stratification or mechanistic validation |
| Probiotics + prebiotics | DED patients, n=41 RCT (Tavakoli et al., 2022/2026) | Oral MULTIBIOTIC™ plus NutriKane D for 4 months | Improved OSDI; NIKBUT and TMH remained stable versus controls | No significant changes in MMP-9, TIMP-1, CRP, or related inflammatory markers | Human RCT evidence | Symptoms, signs, and inflammatory markers were not synchronized |
| Topical postbiotic / oral probiotic | DED patients, n=40 RCT (Heydari et al., 2024) | Triple-masked RCT; L. sakei lysate eye drops and/or oral L. sakei capsules | Topical lysate improved OSDI, TBUT, and Schirmer I; oral capsules showed unclear efficacy | Reduced tear IL-6, TNF-α, and IFN-γ | Single RCT evidence | Efficacy depends on route and formulation |
| IRT5 composite probiotics | Autoimmune or environmentally induced dry eye mouse models | Animal studies using L. casei, L. acidophilus, L. reuteri, B. bifidum, and S. thermophilus | Reduced ocular surface staining and increased tear secretion in autoimmune models; increased tear secretion in environmental models | Increased Treg cells; decreased CD8+IFNγhi cells; altered lacrimal immune proteins and fecal microbiota | Animal mechanistic evidence | Efficacy varies across models; limited human extrapolation |
| S. thermophilus iHA318 | UVB-induced dry eye mice; DES volunteers, n=68 RCT | Animal study plus human RCT | Improved tear volume, TBUT, corneal staining/regularity, OSDI, and tear osmolarity | Antioxidant and anti-inflammatory effects; increased serum sialic acid; reduced NLRP3 activation | Animal evidence + preliminary human RCT | Requires independent cohorts and phenotype-stratified validation |
| Probiotics/prebiotics in metabolic context | Type 2 diabetes-associated dry eye-like mouse model | Animal study using probiotic and prebiotic gavage | Alleviated dry eye-like manifestations and reduced corneal/lacrimal damage | Suppressed TLR4/NF-κB signaling; remodeled gut microbiota; reduced bile acid metabolites | Metabolism-related animal evidence | Human evidence is insufficient |
| Probiotic-derived exosomes/postbiotics | BAC-induced dry eye mice; conjunctival cell models | Preclinical animal and cellular studies of L. fermentum HY7302 and derived exosomes | Improved corneal staining, TBUT, and tear secretion; reduced BAC-induced cellular injury | Downregulated IL-1β, IL-6, IL-8, and MMP-9; modulated AKT/Bcl-2/caspase-3 and tight junction genes | Preclinical mechanistic evidence | No human efficacy or safety validation |
| Perioperative probiotic | FS-LASIK patients, n=100 RCT (Cheng et al., 2026) | Double-blind RCT; oral B. bifidum BB00 during the perioperative period | Reduced postoperative DED incidence; improved OSDI, FBUT, and Schirmer I | 16S rRNA sequencing showed partial reversal of postoperative gut dysbiosis | Human double-blind RCT evidence | Applicable mainly to perioperative settings |
| FMT | Immune-mediated DED/SjS-associated patients, n=10 (Watane et al., 2022) | Open-label clinical exploration using single-donor FMT | No obvious adverse events; 5 patients reported subjective improvement at 3 months | Recipient microbiota shifted toward donor profiles; T-cell profiles correlated with symptoms | Early proof-of-concept evidence | Open-label, small sample, no placebo control |
| Diet-related microbiome modulation | Narrative review/mechanistic synthesis | Mediterranean diet component-based evidence | Possible improvements in TBUT, Schirmer test, and OSDI, but findings are inconsistent | Potential anti-inflammatory, antioxidant, lipid-layer, microbiota-metabolic, and Th17/Treg effects | Indirect mechanistic evidence | Evidence for the overall dietary pattern remains weak |

Abbreviations: BAC, benzalkonium chloride; BUT, tear break-up time; CFU, colony-forming units; CRP, C-reactive protein; DED, dry eye disease; DES, dry eye syndrome; FBUT, fluorescein tear break-up time; FMT, fecal microbiota transplantation; FS-LASIK, femtosecond laser-assisted in situ keratomileusis; IFN-γ, interferon gamma; IL, interleukin; MMP-9, matrix metalloproteinase-9; NIKBUT, noninvasive keratograph break-up time; NLRP3, NOD-like receptor family pyrin domain-containing 3; OSDI, Ocular Surface Disease Index; RCT, randomized controlled trial; SjS, Sjögren syndrome; TBUT, tear break-up time; TIMP-1, tissue inhibitor of metalloproteinase-1; TMH, tear meniscus height; TNF-α, tumor necrosis factor alpha; Treg, regulatory T cells; UVB, ultraviolet B.
